# Medical librarians' knowledge and practices in locating clinical trials for systematic reviews

**DOI:** 10.5195/jmla.2021.1144

**Published:** 2021-04-01

**Authors:** Jennifer C. Westrick, Susan W. Buchholz

**Affiliations:** 1 jennifer_westrick@rush.edu, Library Research Information Specialist, Library of Rush University Medical Center, Rush University Medical Center, Chicago, IL; 2 buchho44@msu.edu, Professor, Associate Dean for Research, Director - PhD Program, Michigan State University, College of Nursing, East Lansing, MI

**Keywords:** systematic reviews, clinical trials, study design methodology, survey

## Abstract

**Objective:**

In regard to locating clinical trials for a systematic review, limited information is available about how librarians locate clinical trials in biomedical databases, including (1) how much information researchers provide librarians to assist with the development of a comprehensive search strategy, (2) which tools librarians turn to for information about study design methodology, and (3) librarians' confidence levels in their knowledge of study design methodology. A survey was developed to explore these aspects of how a medical librarian locates clinical trials when facilitating systematic reviews for researchers.

**Methods:**

In this cross-sectional study, a 21-question survey was sent to medical librarians via several email listservs during April 2020. Respondents were limited to librarians who make the decisions on search terms for systematic reviews.

**Results:**

Responses (n=120) indicated that librarians were often asked to search for various types of clinical trials. However, there was not a consistent method for creating search strategies that locate diverse types of clinical trials. Multiple methods were used for search strategy development, with hedges being the most popular method. In general, these librarians considered themselves to be confident in locating trials. Different resources were used to inform study types, including textbooks, articles, library guides and websites.

**Discussion:**

Medical librarians indicated that while they felt confident in their searching skills, they did not have a definitive source of information about the various types of clinical trials, and their responses demonstrated a clear need and desire for more information on study design methodology.

## INTRODUCTION

Systematic reviews are a synthesized compilation of evidence-based materials, particularly clinical trials, that pertain to a specific clinical question. Librarians who are part of a systematic review team are tasked with locating these materials for the researcher. Librarian involvement in systematic reviews is highly encouraged by several institutions, including The Cochrane Collaboration [1] and the National Academy of Medicine (formerly the Institutes of Medicine) [2].

Librarians who are part of a systematic review team need to understand what the research team is looking for, including whether the research team is seeking specific types of clinical trials [1]. Researcher input about inclusion of clinical trials can be inconsistent and does not always provide the information needed to construct an efficient search strategy. Librarians construct search strings using their knowledge of study design methodology in conjunction with available resources. These available resources include, but are not limited to, hedges (pre-developed search filters that typically consist of both keywords and controlled vocabulary) [3], filters offered by a database, and exclusions (e.g., “NOT”).

Systematic reviews can include many types of studies, which can be challenging to locate. As the Cochrane Handbook for Systematic Reviews of Interventions, Version 6.1, states: “Searching for NRSI [Non-Randomized Studies of Interventions] is less straightforward than searching for randomized trials” [4]. While a basic knowledge of study design methodology is an essential tool for locating clinical trials, there is no definitive resource that provides this information for librarians. A stronger knowledge base about study design methodology would make it more conducive for librarians to include different designs in their search strategies. Also, with a stronger knowledge base regarding different study types, librarians would be able to more quickly and effectively ask researchers questions that would lead to a more precise search for the types of studies the researcher is seeking.

In regard to locating clinical trials for a systematic review, little is known about (1) the information that researchers provide librarians for assistance in developing a comprehensive search strategy, (2) which tools librarians turn to for additional assistance when they search the literature, or (3) librarians' confidence levels in their knowledge of clinical trial types. Therefore, we developed a survey to explore these aspects of how medical librarians locate clinical trials when facilitating systematic reviews for researchers. Specifically, the survey examined researcher input for search strategies, search string development, search strategy confidence, and available resources. To our knowledge, this is the first survey that explores this topic.

## METHODS

### Design

Using a cross-sectional design, a survey (Appendix A) was made electronically available for one month (April 2020).

### Setting and sample

The survey was distributed to seven email listservs that reach medical librarians (Appendix B). All responses were anonymous. Respondents will be called “librarians” for the remainder of this article, and the team of researchers with whom they collaborate will be called “researchers.”

### Measures

After initial development of the survey, three librarian experts and one measurement expert reviewed the survey for accuracy, relevancy, and meaning, and the survey was revised accordingly. The final survey included 21 questions. The first two questions determined whether a potential respondent qualified to participate in the survey. The remaining 19 questions asked about demographic and occupational information (four questions), researcher input (five questions), search string development (two questions), librarian confidence (six questions), and resources (one question), and there was an open-comment question. Questions were answered by using Likert scales, selecting from among multiple choices, and responding to an open-ended format.

### Analysis

RedCap was used to administer the survey and collect results. Excel was used to analyze the data.

### Procedures

The survey was designed to be answered by experienced librarians who work directly with researchers and who make decisions about search terms to include in a literature search for a systematic review. To ensure that this population completed the survey, librarians were required to affirm that they had been credited as a coauthor or acknowledged by name on a systematic review that was published in a peer-reviewed journal. The survey was reviewed and deemed exempt by the Rush University Medical Center Institutional Review Board (IRB ID #20012004-IRB01).

## RESULTS

### Respondent demographics

One hundred twenty librarians completed the survey. It was not possible to calculate a response rate, as members of various listservs may have received the survey more than once. The vast majority of respondents identified as a “Librarian” (92%). Of the remaining respondents, 6% identified as an “Information Specialist” and the remainder identified professionally with other titles. Almost all of the respondents worked in either a university (47%) or an academic or university medical center (36%), while 9% worked in a hospital, 2% in a government setting, and 5% in other settings. The vast majority were from North America (70% from the United States and 11% from Canada), with 17% from Europe, 2% from Australia, and 1% from Asia. Respondents estimated they spent anywhere from 2 to 100 hours creating a search strategy.

### Researcher input on search strategy

**Figure 1:**
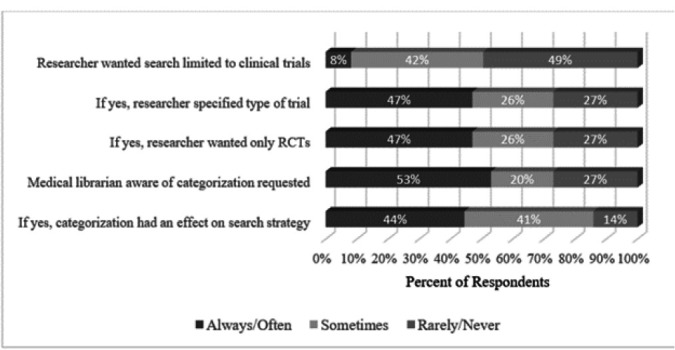
Researchers' input on search strategy

Librarians answered five questions about the type of information provided by researchers when initiating a systematic review (Figure 1). When asked if researchers requested their search strategy be limited to clinical trials, half of librarians said they have received such requests. Of the researchers who made this request, about half specified the types of trials for which the librarian should search (47% reported often or always). Of those researchers who specified they wanted their search limited to trials, fewer than half requested their search be limited to randomized controlled trials (RCTs).

When asked if they were aware of the categorization (e.g., prognostic, causation, therapeutic, guidelines, etc.) of their research, slightly more than half of the librarians (53%) said they always or often knew this information. When asked if this information had an effect on their search strategy, fewer than half (44%) answered always or often.

### Methods for search string development

**Figure 2:**
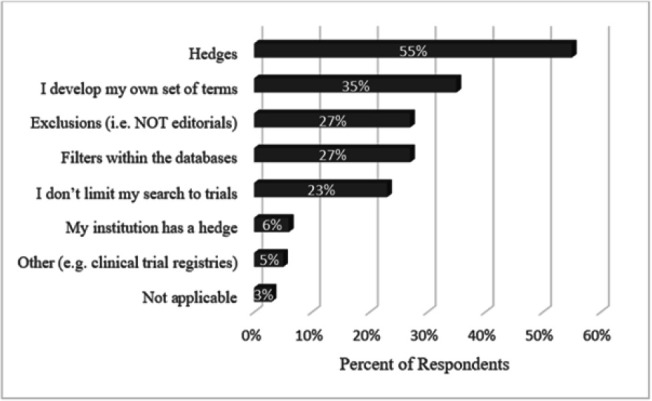
Methods for search string development

When asked to select from among seven popular methods for locating clinical trials (as well as an opportunity to write in a method), librarians revealed there was no one set way to locate them (Figure 2). The top answer was hedges, or pre-formulated search strategies, which were used by 55% of librarians. More than one-third of librarians also developed their own search strings. Other popular answers included using database filters (e.g., PubMed's “Clinical Trials” filter option, used by 27%), keyword exclusions (27%), and simply not searching for trials (23%). Librarians could select more than one method for search string development; 34% chose one method, 22% chose two or three, 13% chose four, and 7% chose five.

### Confidence levels for locating clinical trials

**Figure 3:**
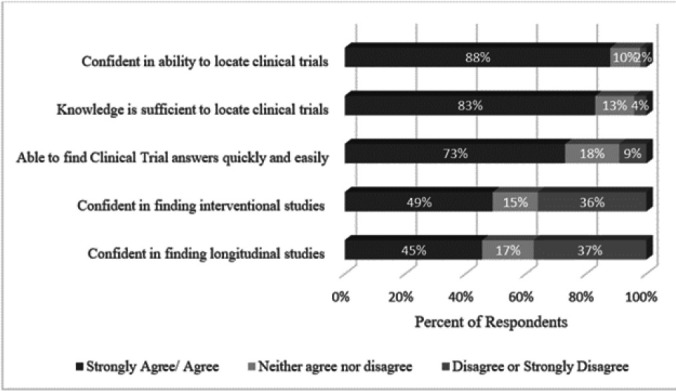
Confidence levels for locating clinical trials

This group of librarians was quite confident in their searching skills, as 45% considered themselves experts, 48% proficient, and 7% competent. No one rated themselves a novice or beginner. Our librarians were also very comfortable locating trials (Figure 3). When asked if they agreed with the statement that they were confident in their ability to locate clinical trials in biomedical databases, 88% agreed or strongly agreed. These expert searchers were also confident in their knowledge of the types of trials themselves. When asked if they agreed that their knowledge of clinical trial types was sufficient to locate such trials for a systematic review, 83% agreed or strongly agreed.

Librarians were asked if they felt they could locate two specific types of clinical trials (interventional or longitudinal) without having to perform research on the types of clinical trials themselves. In response to locating “interventional studies,” 49% agreed or strongly agreed that they could do this without having to conduct research into study types. In response to locating “longitudinal studies,” 45% agreed or strongly agreed that they could do this without having to research study types.

### Resources used for search strategies

**Figure 4:**
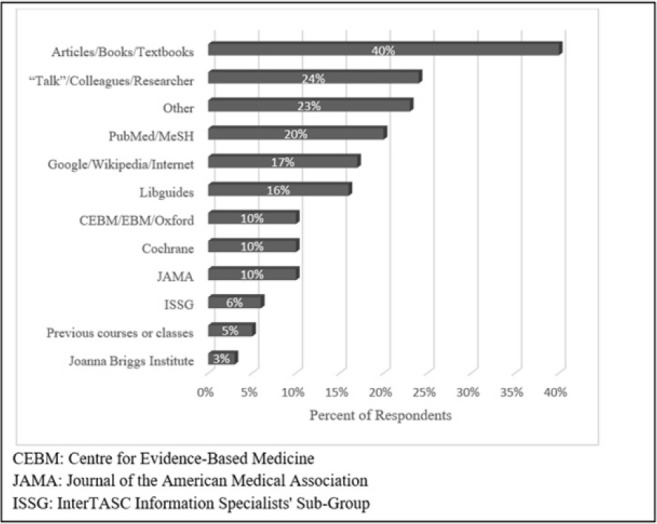
Resources for search strategy development

When asked what resources they turned to for information about study types, 40% of librarians wrote “textbooks” or “articles” with no title or other identifying information (Figure 4). The book or article most commonly cited by name was JAMA's “Users' Guide to the Evidence” [5], which was cited nine times (10%). The next most common resource was talking to colleagues or the researchers (24%). PubMed and MeSH were mentioned by about a fifth of librarians; the next most popular response was the internet, Google, or Wikipedia, with 17% of responses containing one of those words. Almost as popular (16%) was “libguides,” which refers to guides written by other librarians. Industry standards such as the Centre for Evidence-Based Medicine (CEBM) [6], JAMA [5], and Cochrane [1] were each mentioned by 10% of librarians.

## DISCUSSION

Locating clinical trials is often a key element of librarian collaboration on systematic reviews [7]. Half of the survey librarians have been asked at least once to limit a search strategy to clinical trials. Much has been written about how to find randomized controlled trials (RCTs) for systematic reviews [8–10], but not all systematic reviews are based solely on RCTs [11]. Our survey examined librarians' methods, tools, and confidence in locating these various types of trials.

Researchers tended to provide minimal guidance about the types of clinical trials they wanted to see returned by a literature search. Omitted data often included the categorization labels needed to use some hedges. For example, SIGN (The InterTASC Information Specialists' Sub-Group) has a Search Filter Resource that requires a user to choose between categories such as Diagnosis Studies, Epidemiological Studies, or Outcome Studies [12]. Librarians indicated that researchers often did not provide this categorical information, therefore presenting challenges for the librarian who wants to use this type of hedge. Librarians may not want to decide on the correct categorization without the full input of the researcher. As one survey respondent noted:

“Sometimes it seems that a study can have more than one [category]; i.e., prognostic AND diagnostic.”

In addition to hedges, librarians reported using exclusions (e.g., “NOT editorials”), a recognized means of improving the precision of search results [13]. Additionally, almost a quarter of librarians reported simply not including clinical trials in their search strategy. Sometimes this is appropriate, as restricting a literature search to clinical trials may result in the omission of other types of pertinent evidence or primary research.

Other tools for locating clinical trials included databases such as PubMed and the use of internet browsers. Surprisingly, only 10% of librarians mentioned sources widely considered to be reputable in the medical research field, such as Cochrane [1], JAMA [3], or CEBM/EBM/Oxford [6]. The wide range of answers with no single definitive resource as the gold standard for searching for clinical trials revealed that there are many sources of information that are presently being used by librarians, as demonstrated by this survey respondent's comment:

“If I want to revisit a study type … I don't have a single good place to look.”

While, overall, our librarians indicated that they felt very confident in their ability to locate clinical trials and their knowledge of the types of clinical trials, many of these expert searchers did not feel they had sufficient knowledge of specific clinical trial types. It is important to remember that the respondents for this survey are expert searchers; therefore, they are likely not representative of all librarians working on systematic reviews. Even a small lack of confidence or knowledge in this group of expert searchers may indicate that a larger percentage of librarians and others working on systematic reviews are confronting these same challenges. Also, less experienced searchers may need to use informational tools more often.

To further explore confidence levels, two practical examples were provided. When asked if a respondent could find two specific types of trials (interventional and longitudinal), half of the librarians indicated they would need to obtain additional information to more fully understand these types of trial designs. For example, the primary author (JCW) of this article was once asked to locate “all longitudinal studies” for a systematic review and had to conduct additional research to ensure that the search terms for all types of trials that could be considered “longitudinal” were included in the search strategy. Therefore, it could be useful for librarians to encourage the researcher to specify types of trials to ensure accuracy in the intended search.

## LIMITATIONS

To ascertain how librarians search for clinical trials, respondents were limited to librarians who were credited as a coauthor or acknowledged by name on a published, peer-reviewed systematic review. This was done to ensure that the survey was completed by librarians who work closely with researchers and make decisions about which terms to include in systematic review search strategies. While this approach yielded targeted information about the decision-making process of experienced librarians, it is not necessarily representative of all librarians who work on systematic reviews. Another limitation is the size of the respondent pool, as the completed responses of 120 respondents may not be representative of the entire population of librarians who collaborate on published systematic reviews. Also, responses were collected in April 2020, a time when many librarians were shifting to a remote work location due to the COVID-19 pandemic, which may have affected the response rate.

This survey focused on locating published articles about clinical trials and did not ask about searching trial registries such as ClinicalTrials.gov [14]. Scoping reviews often include clinical trials as well as other types of material. This survey focused on systematic reviews only and perhaps missed some information about locating clinical trials that could have been found by including scoping reviews in the methodology. Protocols for upcoming systematic reviews are often submitted to registration sites such as PROSPERO [15] or the Center for Open Science (OSF) [16]. These protocols often include information about the types of clinical trials for which researchers are searching. This survey did not ask about librarian involvement in, or knowledge of, such protocols.

## CONCLUSION

The expert librarians who responded to our survey indicated that collaborating on systematic reviews often involves locating clinical trials. While they feel confident in their searching skills, they do not have a definitive source for information about the various types of clinical trials, and their comments demonstrate a clear need and desire for this information. Future research efforts might include an examination of curricula in popular systematic review courses regarding study design methodology. One respondent noted:

“I really want to take a class on study design methodology. If there were a class/webinar like this—study design methodology for librarians designing search strategies for SRs [systematic reviews]—I'd take it in a heartbeat!”

## Data Availability

Data associated with this article are available in FigShare at https://figshare.com/articles/dataset/Abstract_and_Survey_With_Results_docx/13469529.

## SUPPORTING AGENCIES

The author was a fellow in the MLA's Research Training Institute (RTI), which was funded in part by a grant from the Institute of Museum and Library Services (RE-95-17-0025-17).

## SUPPLEMENTAL FILES

**Appendix A:** Exploring How Medical Librarians Locate Clinical Trials for Systematic Reviews Survey

**Appendix B:** List of the Seven Listservs That Received the Emailed Invitation to Participate in the Survey
