# Supplementary material for: Medical librarians' knowledge and practices in locating clinical trials for systematic reviews
Source: J Med Libr Assoc. 2021 Apr 1;109(2):295–300. doi: 10.5195/jmla.2021.1144 (PMC8270353; doi:10.5195/jmla.2021.1144)
Supplement: Supplementary file 1 — Appendix A: Exploring How Medical Librarians Locate Clinical Trials for Systematic Reviews Survey [file jmla-109-2-295-s01.docx]

| **Exploring how Medical Librarians Locate Clinical Trials for Systematic Reviews Survey**  **Respondent Qualification**   1. Do you conduct literature searches for systematic reviews? (If Yes, answer #2; if No, you have completed the survey) 2. Have you been credited as a co-author or acknowledged by name in a peer-reviewed publication for your participation in conducting the literature search of a systematic review? (If Yes, proceed; if No, you have completed the survey)   **I. Demographics/Occupation**   1. Which of the following best describes your job? Choose one. (Librarian, Informationist, Other) 2. How would you describe the place where you work? _______ 3. In what country do you work? 4. While every systematic review is different, how many hours would you estimate that you typically put into creating the search strategy for a systematic review? ____   **II. Researcher Input Provided for Search Strategy**  Thinking of the systematic reviews you’ve worked on and in which you were credited as a co-author or acknowledged by name in a peer-reviewed publication (always, often, sometimes, rarely, never):   1. When discussing the literature search, how often did the researchers specify that they want the search results *limited to clinical trials*? 2. If the researcher said they wanted results limited to trials, how often did the researchers specify the *types* of trials (i.e. randomized, interventional, etc.) for which you should search? 3. If the researchers specified types of trials, how often did the researchers want you to locate *only randomized controlled trials*? 4. Research is sometimes put into categories such as prognostic, causation, therapeutic, etiology, guide-lines, etc. How often are you aware of such a categorization for a research project you are working on? 5. If you are aware of such categorization, does it affect your search strategy?   **III. Search String Development**   1. If the researcher wants you to search for clinical trials (of any type), how do you locate these trials?   Click **all** that apply.   - 1. I use a pre-formulated search filter (i.e. a hedge)   2. I use filters offered by the databases   3. I develop my own set of terms   4. My institution has a recommended set of terms   5. I didn’t limit my search to trials; instead I ask the researchers to look for trials in the title/abstract   screening process   - 1. I use exclusions (i.e. NOT editorials)   2. Not applicable; researchers never ask me to limit search results to clinical trials   3. Other:  1. If you use search filters (even if just occasionally), what are some of the pros and cons? (fill-in-the-blank)   **­­­**  **IV. Search Strategy Confidence**   1. How would you describe your searching skills? (novice, advanced beginner, competent, proficient, expert)   How confident are librarians that they have the skills needed to effectively conduct literature searches for systematic reviews? (strongly agree, agree, neither agree nor disagree, disagree, strongly disagree)   1. I am confident in my ability to locate clinical trials in the biomedical databases (i.e. PubMed). 2. My knowledge of the types of clinical trials is sufficient to locate them for a systematic review. 3. When I have questions about types of clinical trials, I can find the answers quickly and easily 4. If a researcher asked me to “find all interventional studies” I could do that without having to do research into the study types I've been asked to search 5. If a researcher asked me to “find all longitudinal studies” I could do that without having to do research into the study types I've been asked to search   **V. Search Strategy Resources**   1. If you do seek information about study types, what resources do you use? (fill-in-the-blank – check if correct)   **Comment from Respondent**   1. Any other thoughts on finding clinical trials for systematic reviews? |
| --- |
