# Supplementary material for: Medical librarians' knowledge and practices in locating clinical trials for systematic reviews
Source: J Med Libr Assoc. 2021 Apr 1;109(2):295–300. doi: 10.5195/jmla.2021.1144 (PMC8270353; doi:10.5195/jmla.2021.1144)
Supplement: Supplementary file 2 — Appendix B: List of the Seven Listservs That Received the Emailed Invitation to Participate in the Survey [file jmla-109-2-295-s02.docx]

List of the seven listservs that received the emailed invitation to participate in the survey

1. AAHSL (Association of Academic Health Sciences Libraries) listserv
2. ACRL’s (Association of College and Research Libraries) Systematic Reviews and Related Methods Interest Group listserv
3. MLA’s (Medical Library Association) Clinical Librarians and Evidence-Based Healthcare Caucus listserv
4. MLA’s Expert searching listserv
5. MLA’s Health Association and Corporate Libraries Caucus listserv
6. MLA’s NAHRS (Nursing and Allied Health Resources Caucus) listserv
7. MLA’s Systematic Review Caucus listserv
